# An organic brain-inspired platform with neurotransmitter closed-loop control, actuation and reinforcement learning†

**DOI:** 10.1039/d3mh02202a

**Published:** 2024-04-30

**Authors:** Ugo Bruno, Daniela Rana, Chiara Ausilio, Anna Mariano, Ottavia Bettucci, Simon Musall, Claudia Lubrano, Francesca Santoro

**Affiliations:** a Tissue Electronics, Istituto Italiano di Tecnologia 80125 Naples Italy; b Dipartimento di Chimica, Materiali e Produzione Industriale, Università di Napoli Federico II 80125 Naples Italy; c Institute of Biological Information Processing – Bioelectronics, IBI-3, Forschungszentrum Juelich 52428 Germany; d Neuroelectronic Interfaces, Faculty of Electrical Engineering and IT, RWTH Aachen 52074 Germany; e Faculty of Medicine, Institute of Experimental Epileptology and Cognition Research, University of Bonn, Bonn Germany

## Abstract

Organic neuromorphic platforms have recently received growing interest for the implementation and integration of artificial and hybrid neuronal networks. Here, achieving closed-loop and learning/training processes as in the human brain is still a major challenge especially exploiting time-dependent biosignalling such as neurotransmitter release. Here, we present an integrated organic platform capable of cooperating with standard silicon technologies, to achieve brain-inspired computing *via* adaptive synaptic potentiation and depression, in a closed-loop fashion. The microfabricated platform could be interfaced and control a robotic hand which ultimately was able to learn the grasping of differently sized objects, autonomously.

## Abstract 2

Organic neuromorphic platforms have recently received growing interest for the implementation and integration of hybrid systems, acting as a bridge between biological tissue and artificial computing architectures.

## Graphical abstract

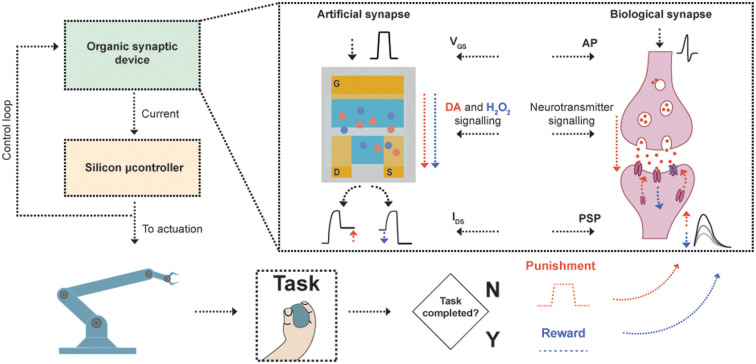

**New concepts**

In this manuscript a brain-inspired closed-loop system has been demonstrated for the accomplishment of motor control and actuation tasks, through a learning process mediated by a neurotransmitter. This system integrates well established silicon technologies with organic materials, more suitable for communication with biological neurons. While the intelligence and the decision-making process is completely delocalized, it lies in the local adaptation of a neuromorphic OECT. Such adaptation is closed-loop controlled using a PID control law, and it mimics the neurotransmitter-mediated synaptic plasticity of biological neural networks (BNNs). From the existing field of research, sensing and motion control have been achieved by exploiting organic neuromorphic architectures, with the goal to recapitulate autonomous local learning typical of the human neural processing (Krauhausen, I. *et al.* Organic neuromorphic electronics for sensorimotor integration and learning in robotics. *Science Advances***7**, eabl5068, 2021). In these applications there are two main aspects still missing: the first one is the lack of a neuromorphic control-loop architecture strongly desirable for the adaptive responsiveness to external stimuli of the system; the second one is the employment of biological signalling as responsible of the synaptic plasticity during the learning process, typical of the human brain and useful for the active integration of this technologies in a biological environment. In this new concept dopamine is the signal used for the strengthen of the artificial synapse, integrated in a closed-loop system able of adaptive and reinforcement learning with an object-specific recognition and training.

## Introduction

Neuromorphic computing represents an emerging and promising approach in the development of the next generation of hardware. The continuous endeavour of researchers reaches a panoply of different fields, spanning from the design of digital processing architectures, to the development of smart adaptive hardware. In this sense, organic semiconductors, featuring a polymer structure and ionic-electronic mixed conduction properties, have been shown to emulate short- and long-term memory^1–3^ when integrated into three terminal devices such as organic electrochemical transistors (OECTs) and arrays.^4–6^ Here, these devices were capable of information processing^7,8^ classification,^9,10^ performing reservoir computing,^11,12^ act as fast switching memory elements^13^ and spiking neuronal networks.^14–17^ Furtherly, inorganic materials have been largely employed to engineer artificial neurons,^18^ with the aim of improving device responsiveness, lowering power consumption and integrating into large arrays. On the other hand, organic materials have recently found major applications as neuromorphic interfaces.^19^ In fact, conductive polymers can also provide a biocompatible and stable coupling for biological cells and tissues and therefore support hybrid interfaces between artificial neuromorphic and living neuronal systems.^20,21^ Notably, neurohybrid synaptic platforms have been built exploiting electrical communication,^22^ electrochemical signalling through single and multiple neurotransmitter release^23,24^ and direct tissue interfacing and stimulation with implantable probes.^25^

In addition, neuromorphic architectures, by exploiting neural primitives, may enable real-time interaction with the surrounding environment. Indeed, state-of-the-art neuromorphic controllers may either leverage on neuromorphic models to determine a control law,^26,27^ or they can rely on spiking neural networks (SNNs) to implement long-standing control laws, such as proportional, integral and derivative (PID) controllers on silicon neuromorphic chips.^28,29^ Crucially, such approaches still fail in recapitulating the autonomous adaptation that characterizes neural processing. As a result, while neuromorphic sensing and motion control in organic neuromorphic architectures were demonstrated,^7,30^ a neuromorphic control-loop controlled system is still missing. Indeed, the implementation of a neuromorphic closed-loop architecture is strongly desirable, enabling fully autonomous systems, that could adapt as a response to external stimulation. Here, we present a simple and direct approach in the realization of a neuromorphic closed-loop system, in which silicon and organic materials cooperate to accomplish real world tasks, in real-time. In the presented closed-loop system a silicon microcontroller controls actuation and motion, while the intelligence and the decision-making process is completely delocalized, as it lies in the local adaptation of a neuromorphic OECT. Such adaptation is closed-loop controlled using a PID control law, and it mimics the neurotransmitter-mediated synaptic plasticity of biological neural networks (BNNs). Dopamine (DA) oxidation was exploited to strengthen/weaken the artificial synapse, that controls in real time the opening and closure of a robotic hand in a closed-loop configuration. Finally, autonomous reinforcement learning, based on a reward/punishment protocol is implemented. Here, the organic synaptic device ‘learns’ how to drive the hand to grasp different-sized object. The proposed architecture demonstrated how an organic brain-inspired platform can cooperate with either biological and electronic systems, establishing a neuromorphic closed-loop system.

## Results and discussion

**Fig. 1:**
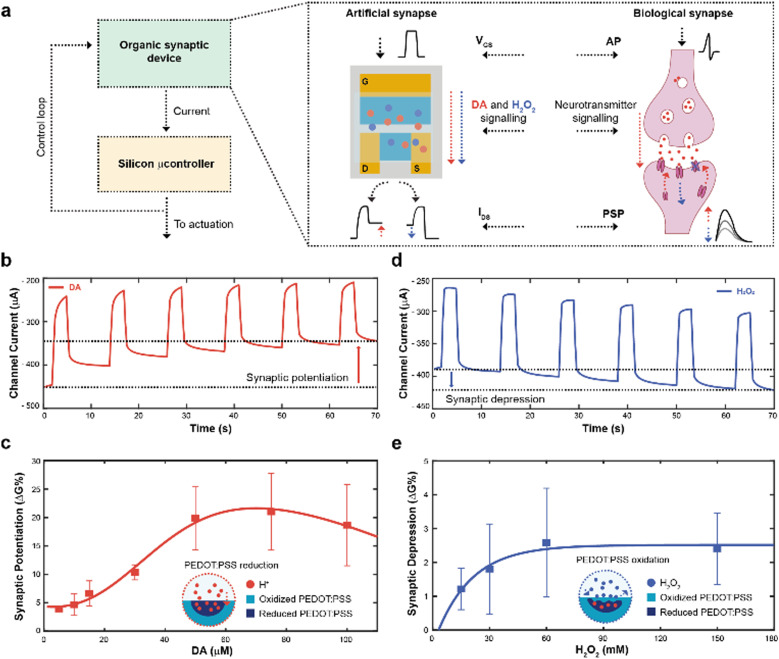
Overall neuromorphic closed-loop architecture and open-loop artificial neuron characterization. (a) Schematic of a closed-loop neuromorphic system, coupling a silicon microcontroller to drive external actuation, while delocalizing the decision-making process to the organic synaptic device (left). Comparison between a biological synapse and the organic synaptic device (right). (b) Synaptic potentiation elicited by a train of square voltage pulses applied at the gate terminal in presence of DA. (c) Synaptic potentiation as a function of DA molarity (Numerical values: 3.9 ± 0.3; 4.6 ± 1.9; 6.6 ± 2.2; 10.3 ± 1.3; 19.8 ± 5.5; 21.0 ± 6.7; 18.7 ± 7.1; (*N* = 3)), along with schematic of the PEDOT:PSS reduction mechanism. (d) Synaptic depression elicited by a train of square voltage pulses applied at the gate terminal in presence of H_2_O_2_. (e) Synaptic depression as a function of H_2_O_2_ molarity (Numerical values: 1.2 ± 0.6; 1.8 ± 1.3; 2.6 ± 1.6; 2.4 ± 1.0; (*N* = 3)), along with a schematic of the PEDOT:PSS oxidation mechanism.

The working principle of the neuromorphic device is schematically represented in Fig. 1a. The channel current of the electrochemical neuromorphic organic device (ENODe) was sampled and digitalized, enabling the communication with inorganic hardware, that would eventually drive an actuation.

In brief, the neural signalling was recapitulated by applying square voltage pulses at the gate terminal, emulating action potentials (APs) of biological synapses. In a biological synapse, when an action potential reaches the axon terminal of the pre-synaptic neuron, neurotransmitters are released in the synaptic cleft. Such neuroactive molecules, then, bind to specific receptors of the post-synaptic neuron, eliciting an electrical response.^31^

In the organic synaptic device, upon the application of the gate potential, ions would migrate from the electrolyte to the polymeric channel of the transistor, resembling the neurotransmitter release. In addition, such ions would dope/de-dope the polymeric channel of the transistor, modulating its current, and mirroring the post-synaptic potential response.

In addition, continuous stimulation/inhibition of neural connections results in potentiation/depression of specific neural pathways, resulting in the so-called synaptic plasticity.^31^ Here, the ENODe could emulate such synaptic potentiation and depression, by controlling in real time the local concentration of a neurotransmitter. A faradaic reaction was indeed exploited to elicit a charge transfer mechanism, doping/de-doping the channel of the ENODe in a non-volatile way (Fig. 1b, c and d, e, respectively).

Among the variety of voltage-oxidizable neurotransmitters^23,32^ that could be employed in the non-volatile conductance modulation of the ENODe channel, dopamine (DA) was chosen, as this molecule is crucial in the closed-loop circuitry of motor-learning in the brain, in which it is used to enforce positive behavioural outcomes, such as the correct execution of a movement.^33,34^

First, open-loop non-volatile potentiation and depression (*i.e.*, synaptic plasticity) of the ENODe were demonstrated and characterized. When DA was present in the electrolyte, the application of a gate bias, matching the neurotransmitter's oxidation potential,^32^ favoured an oxidation reaction and a consequent release of protons and electrons in the electrolyte.^35^ Cations generated in such reaction would elicit a charge-transfer process to both gate and channel of the organic transistor, reducing the PEDOT:PSS and finally decreasing the device's conductance (Supplementary Discussion S1, ESI†).^24^ As a result, the redox reaction permanently de-doped the ENODe channel, as the current decreased with the number of pulses (Fig. 1b). The change in the baseline of the current (Fig. 1b) represented the synaptic potentiation of the neuromorphic device, as it mirrored the long-term strengthening of biological synapses that takes place as a response to an increased stimulation.^36^

Notably, this was a concentration-dependent process (Fig. 1c). A linear dependence of the synaptic potentiation on the concentration of DA employed in the faradaic process was observed in the range 5–30 μM. However, at higher values (50 to 100 μM), the channel conductance modulation would exhibit a saturation behaviour without any significant increase (Fig. S1, ESI†). Here, protons released during the DA oxidation reaction penetrated the bulk of the CP (Fig. 1c), progressively reducing the PEDOT:PSS and de-doping the transistor channel^24^ whereas the saturation behaviour might occur because of the limited oxidative species present at the gate electrode.^23^

In addition, synaptic depression could be recapitulated here by introducing H_2_O_2_ in the electrolyte solution (Fig. 1d) that oxidizes PEDOT:PSS, reversing the DA-mediated (30 μM) de-doping, restoring the initial conductance level of the ENODe (Supplementary Discussion S1, ESI†).

As shown in Fig. 1e, the conductance variation was not dependent on the concentration of H_2_O_2_: the synaptic depression slightly increased with increasing H_2_O_2_ concentration (up to 60 mM), while it remained constant for higher concentrations. Such non-dependency of the synaptic depression on the concentration of the analyte may imply that the H_2_O_2_-mediated oxidation of PEDOT:PSS was elicited by a reaction occurring at the surface of the material (Fig. 1d; Fig. S2, ESI†).^37^ Importantly, the difference in the numerical values of synaptic potentiation and depression was correlated to the aforementioned PEDOT:PSS reduction and oxidation mechanisms. In the former, the oxidation of the neurotransmitter could immediately reduce the polymer, dramatically reducing its conductance. In the latter, the H_2_O_2_ in the electrolyte slowly oxidized the PEDOT:PSS, recovering its conductance.

**Fig. 2:**
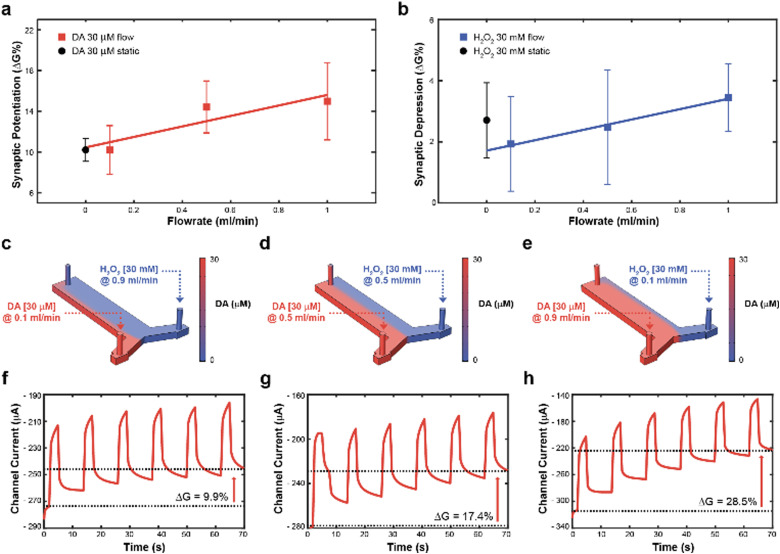
Artificial neuron closed-loop application and synaptic plasticity control. (a) Synaptic potentiation as a function of the flowrate when 30 μM DA was present in the electrolyte (red trace, numerical values: 10.2 ± 2.4; 14.4 ± 2.5; 15.0 ± 3.8; (*N* = 3)) and comparison with synaptic potentiation obtained with 30 μM DA under static condition (black circle. Numerical values: 10.3 ± 1.3; (*N* = 3)). (b) Synaptic depression as a function of the flowrate when 30 mM H_2_O_2_ was added to the electrolyte (blue trace. Numerical values: 1.9 ± 1.5; 2.5 ± 1.9; 3.4 ± 1.1; (*N* = 3)) and comparison with synaptic potentiation obtained with 30 mM H_2_O_2_ under static condition (black circle. Numerical values: 1.8 ± 1.3; (*N* = 3)). (c)–(e), Numerical simulation of DA and H_2_O_2_ distribution inside the microfluidic channel at different flowrates ((c), 30 μM DA, 0.1 ml min^−1^ and 30 mM H_2_O_2_, 0.9 ml min^−1^; (d) 30 μM DA, 0.5 ml min^−1^ and 30 mM H_2_O_2_, 0.5 ml min^−1^; (e) 30 μM DA, 0.9 ml min^−1^ and 30 mM H_2_O_2_, 0.1 ml min^−1^), along with numerical simulation of the total DA concentration in the microfluidic channel. (f)–(h), channel current modulation obtained in the same combinations of simulated 30 μM DA and 30 mM H_2_O_2_ flowrates ((c), (d), and (e), respectively).

Subsequently, to achieve real time control of the synaptic potentiation/depression, a *y*-shaped microfluidic module (see Methods) was coupled to the transistor to regulate the DA and H_2_O_2_ – gate interface and therefore exploit the flowrate as a control variable. In fact, as previously reported, the flowrate might increase the number of species available for the oxidation, while preventing fouling at the gate electrode.^23^ While no significant difference in synaptic potentiation was observed for a 30 μM DA solution under static condition and at low flow rates (0.1 ml min^−1^), high flow rates (0.5 and 1 ml min^−1^) induced an almost linear increase of the conductance modulation (Fig. 2a). Furthermore, a linear dependence of synaptic depression was observed when employing the H_2_O_2_ solution at different flow rates, suggesting that the flowrate increases the amount of solution that actively washed the surface of the polymeric channel (Fig. 2b).

Then, to determine the simultaneous effect of DA and H_2_O_2_ solutions on the synaptic modulation, different flow rates conditions were numerically simulated (Fig. 2c–e) and correlated to the measured channel conductance variation (Fig. 2f–h). Notably, different distributions of DA and H_2_O_2_ were achieved in the microfluidic channel by changing the ratio between flowrates 
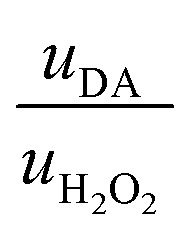
. When *u*_DA_ ≪ *u*_H_2_O_2__, (Fig. 2c)

Thus, the DA-mediated synaptic potentiation (synaptic potentiation Δ*G* = 9.9%, Fig. 2f) was comparable to the potentiation obtained with same concentration of neurotransmitter under static conditions (Fig. 1c). When 
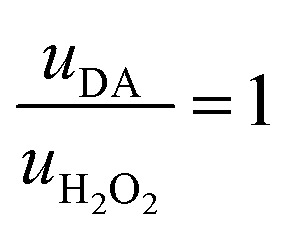
, half of the microfluidic channel volume was filled with DA, increasing the synaptic potentiation in comparison to the static case (Δ*G* = 17.4%, Fig. 2g). Lastly, as *u*_DA_ ≫ *u*_H_2_O_2__, DA was present in the whole microfluidic channel, strongly increasing the synaptic potentiation (Δ*G* = 28.5%, Fig. 2h).

**Fig. 3:**
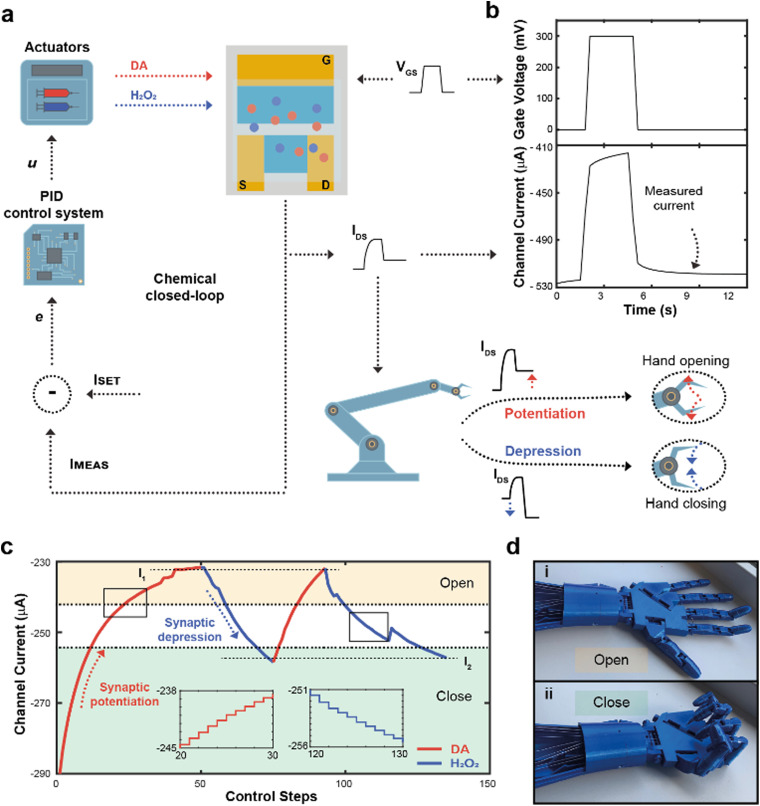
Closed-loop neuromorphic system and robotic actuation. (a) Block diagram of the closed-loop neuromorphic system. *I*_DS_ was measured and compared to a desired setpoint, computing the error of the closed-loop system *e* and then the control law *u*, subsequently releasing either DA or H_2_O_2_, changing their concentration inside the electrolyte of the ENODe, ultimately closing the loop. In addition, *I*_MEAS_ was digitalized and sent to the robot, opening or closure the hand. (b) Square voltage pulse applied at the gate terminal during the closed-loop operation, along with the measured current. (c) Closed-loop regulation of the channel current of the neuromorphic device, connected to the robotic hand. Two setpoints (*I*_1_, and *I*_2_, horizontal dotted lines) were chosen as - 235 and - 260 μA, respectively. If the measured current was higher than the setpoint, DA was released to potentiate the synaptic device (red trace), decreasing the amount of current flowing inside the polymeric channel. Conversely, when *I*_MEAS_ was lower than *I*_SET_, H_2_O_2_ was released in the electrolyte (blue trace) achieving synaptic depression. In addition, two tasks were identified (open and closed hand, yellow and green regions, respectively), corresponding to different neurotransmitter's level. As *I*_MEAS_ reached the ‘open’ region, the hand is completely open. Conversely, when the current was within the ‘close’ region, the hand was fully closed. (d) Pictures of the open (d-i) and closed hand (d-ii) during the closed-loop measurements.

The flow rate could therefore be selected as a control parameter to regulate the long-term modulation of the ENODe and a closed-loop system including an organic synaptic device was implemented (Fig. 3a).

Here, the completion of a desired task (*i.e.*, hand closure/opening) corresponded to a certain channel current (setpoint), and the competing DA/H_2_O_2_ flow rates were closed-loop controlled to reach this setpoint. At each loop iteration, the channel current was measured (*I*_MEAS_) and compared to the setpoint (*I*_SET_), to quantify the error (*e* = *I*_SET_ – *I*_MEAS_) of the closed-loop system. This error described how far the actual value of current was from the setpoint. At this stage, a PID controller interpreted the error and computed a control law *u*, as a linear combination of the error, its integral and derivative over time (Fig. S3, ESI†).

The control law *u* determined the optimal flowrates of DA and H_2_O_2_ to minimize the error *e*. Finally, a square voltage pulse was applied at the gate terminal (*V*_GS_), oxidizing DA (if present in the microfluidic channel), changing the channel conductance and, consequently, the channel current. The current was measured again, determining a new value of *I*_MEAS_, and triggering a new iteration. This process was repeated until the channel current reached the setpoint (*e* < *ε*).

Notably, the channel current I_DS_ was measured after the application of a pulse (Fig. 3b), not including the transient response of the transistor due to ions injected from the electrolyte to the polymeric channel (rising edge of *V*_GS_) and then migrating back to the electrolyte (falling edge of *V*_GS_).^38^

In addition, the value of *I*_MEAS_ was sent to a microcontroller that determined a control signal as an input to servo motors, driving a 3D-printed robotic hand (Fig. S4, ESI†).

The robotic hand could be closed or opened by adjusting *I*_SET_, transducing synaptic potentiation/depression of the ENODe into motor commands: initially, (*i.e.*, before execution of the closed-loop system) the value of *I*_MEAS_ sent to the microcontroller was encoded as a complete closure of the hand. If, depending on *I*_SET_, synaptic potentiation occurred, *I*_MEAS_ progressively decreased. Such current decrease would be encoded by the microcontroller as the opening command of the robotic hand. Conversely, in case of synaptic depression, the *I*_MEAS_ increase would be encoded as a closing command of the hand.

Fig. 3c shows a closed-loop system example where two setpoints were arbitrarily chosen (*I*_1_ and *I*_2_, dashed horizontal lines). Initially, as *I*_SET_ was lower than *I*_MEAS_, the system required synaptic potentiation to complete the regulation task. DA was released inside the electrolyte (Fig. 3c), while square voltage pulses were applied at the gate terminal, oxidizing the neurotransmitter, and decreasing the ENODe conductance by reducing PEDOT:PSS. Concurrently, the prosthetic hand gradually opened as *I*_MEAS_ decreased, until full opening was achieved (Fig. 3d). Eventually, the measured current *I*_MEAS_ decreased, and the regulation task was successfully completed, *i.e.*, *I*_MEAS_ ≅ *I*_SET_. Once the value *I*_1_ was reached, *I*_SET_ changed to *I*_2_. Here, as the setpoint was higher than the measured current, the system required synaptic depression. The control system responded to such variation by releasing H_2_O_2_ (Fig. 3c, blue trace), oxidizing the surface of PEDOT:PSS and increasing the channel current, that reached the setpoints (Video S1, ESI†). The workflow of the software for the control is reported in Supplementary Discussion S2 (ESI†).

**Fig. 4:**
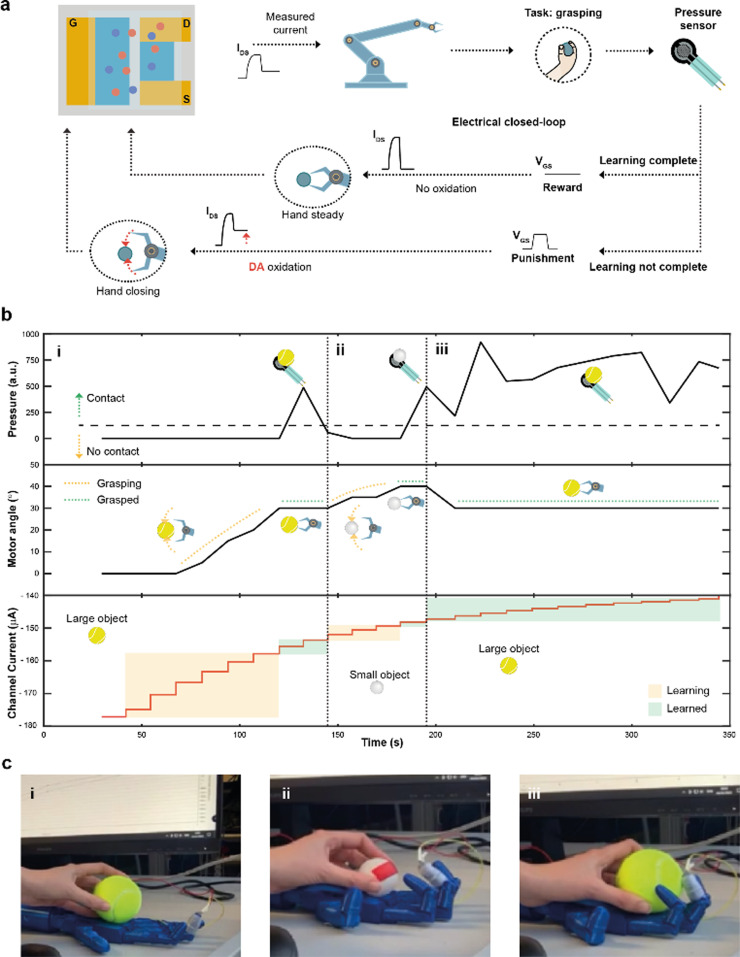
Closed-loop reinforcement learning. (a) Block diagram of the electrical closed-loop for autonomous reinforcement learning. The measured current *I*_MEAS_ was digitalized, controlling the grasp of the hand, that was equipped with a pressure sensor. Based on the sensor readout, it was possible to determine either if the hand was able to complete (or not) a task, *i.e.*, grasping an object. In case of failure, DA was released in the electrolyte solution and a punishment electrical signal (square voltage pulse) was applied at the gate terminal, decreasing *I*_MEAS_ and further closing the hand. In case of a successful execution of the task, a reward signal was applied (*V*_GS_ = 0 V), while removing DA from the electrolyte. (b) Measurements of the electrical closed-loop during a reinforcement learning experiment. (b-i), A tennis ball was employed. If the pressure sensor did not detect the contact with the object (top plot), DA was continuously oxidized in the microfluidic channel, decreasing the channel current (bottom plot) and closing the hand by increasing the angles of the motors (middle plot). The procedure stopped when the grasping is complete. (b-ii) A ping-pong ball was introduced into the system, restarting the learning procedure. (b-iii) The former object (tennis ball) was presented again to the system. Here the grasp was completed instantaneously, as the ENODe had already learned to grasp such object. (c) Pictures of the hand grasping a large object (tennis ball, (c-i)), a small object (ping pong ball, (c-ii)) and the larger object once again (c-iii).

Finally, by integrating a pressure sensor in the closed-loop architecture (Fig. 4a), reinforcement learning^39^ was introduced. The goal of this system was to learn to recognize and grasp objects of different sizes, using reward and punishment signals, as shown in Fig. 4a.

The task presented increased because of the variable object size where diverse degrees of movement were required to adjust and close the hand motors. Initially, the hand was completely open and the current *I*_MEAS_ determined the closure of the robotic hand. The extended system could sense the environment through the sensor, describing whether the hand was able to grasp the object or not. In case of a failed grasp, a punishment signal in the form of a square voltage pulse was applied at the gate terminal, oxidizing DA present in the electrolyte of the device. The punishment signal caused *I*_MEAS_ to decrease, leading to a further closure of the hand. Conversely, when the hand was able to grasp the object, a reward action was provided, by biasing the gate terminal at zero voltage level, keeping a stable channel current, and preventing a further closure of the hand.

A large (tennis ball) and small (ping pong ball) objects were used in the reinforcement learning experiment. The pressure was continuously read from the sensor to detect contact with the test object, while the angle of the motors was recorded and used as a hand closure parameter. The channel current (measured after each reward/punishment signal), instead, indicated the learning of the neuromorphic ENODe (Fig. 4b).

First, the tennis ball was employed (Fig. 4b-i), and the robotic hand closed (based on the value of *I*_MEAS_) in the attempt of grasping it. The task initially failed (Fig. 4c-i) and consequently a punishment signal was supplied, oxidizing DA, and decreasing *I*_MEAS_. As this process was iterated, the oxidation of the neurotransmitter progressively caused the hand to close (increasing motor angle). When the ENODe learnt how to grasp the tennis ball (*i.e.*, when the pressure sensor detected that the hand correctly touched the object), the reward was provided, and the robot stopped moving (constant motor angle). Then, the ping pong ball was introduced (Fig. 4b-ii). Because the closed-loop system was not trained to grasp such a small object (Fig. 4c-ii), the hand failed in executing the assigned task. Therefore, a punishment signal was supplied, eliciting DA oxidation, and progressively closing the hand (motor angle increases). Punishment signals were continuously provided until the hand completely grasped the smaller ball and completed the learning protocol. Further details are reported in Fig. S5 (ESI†).

Last, the first object (tennis ball) was introduced again. Here, considering that the ENODe had already learned how to grasp it before, the object was immediately grabbed correctly (Fig. 4b-iii and 4c-iii and Video S2, ESI†).

## Experimental methods

### Device fabrication

ENODe were fabricated on a 25 × 25 mm square glass substrate, with 10 × 10 mm indium tin oxide (ITO) square at each corner (Xinyan Technology Ltd, QGM20210930119). PEDOT:PSS (Hereaus, Clevios PH1000, 81076212) aqueous solution was prepared by adding 5 vol% ethylene glycol (Sigma-Aldrich, 102466-1L-M), 1 vol% (3-glycidyloxypropyl)trimethoxysilane (Sigma-Aldrich, 440167-100ML) and 0.02 vol% dodecylbenzene sulfonic acid (Sigma-Aldrich, 44198-250ML). Glass substrates were treated with oxygen plasma (Tecno-Service) for 2 min at 20 W. Subsequently the PEDOT:PSS solution was spin coated on the substrate at 2000 rpm for 2 min. Thermal annealing at 140 °C on hotplate was performed. PEDOT:PSS gate and channel were patterned through oxygen plasma dry etching technique for 15 min, at 100 W. The physical masks used to define the transistor geometry during the etching procedure were made of polydimethylsiloxane, mixed in ratio 10 : 1 wt/wt with a crosslinker, and cured at 80 °C, 1 h (PDMS, Silgard 184). Finally, through the etching process two symmetrical PEDOT:PSS stripes 7 × 17 mm wide were deposited 2 mm apart. Then the devices were immersed in milliQ water for 1 h to allow for the complete swelling of the PEDOT:PSS prior to further measurements.

### Microfluidic system

To allow the electrolyte to flow between the channel and the gate of the ENODe, a microfluidic channel, made of PDMS, was attached on the device by means of uncured PDMS, placed on the edges of the channel, and subsequently cured at 80 °C, 1 h. The microfluidic channel had a rectangular shape (17 × 4 mm) with a *y*-shaped junction at one end. Three holes were created with the use of a 1.2 mm punch at the ends of the *y*-shaped channel. Two input holes were connected through microfluidics tubes to two pumps, one for the DA solution and the other one for the H_2_O_2_ solution. The third output hole was connected to a waste container.

### Electrolyte solution preparation

PBS without Ca^++^ and Mg^++^ buffer solution was purchased from Life Technologies, 14190169. DA solution was obtained by dissolving dopamine hydrochloride in powder (Sigma-Aldrich, USA) in PBS. A stock H_2_O_2_ (30% in water, Sigma-Aldrich, USA) was diluted in PBS to obtain H_2_O_2_ solutions.

### Electrical measurements

All electrical measurements were carried out using a commercially available setup (Arkeo, Cicci Research, Italy) featuring two independent source measure units (SMUs) simultaneously. Pulsed operations were performed by keeping a fixed bias voltage *V*_DS_ = −0.2 V, while applying square voltage pulses *V*_GS_ at the gate terminal. Each measurement consisted of 6 pulses with amplitude 0.3 V, pulse width 3 s and delay between pulses 9 s.

### PID control system

The PID control system was embedded in a LabVIEW routine running on the measurement setup. The proportional coefficient is set to 0.005, the integral one was set to 0.05, while the derivative was set to 0.01. These parameters were found through manual PID calibration. All the details for the software were reported (Supplementary Discussion S2, ESI†).

### DA static measurements and data analysis

100 ml DA solution was inserted in the microfluidic coupled to the channel of the ENODe. Each measurement consisted of 6 voltage pulses applied at the gate terminal. After each measurement three washes were performed with 100 ml of fresh PBS solution. The channel conductance was calculated by dividing the channel current by *V*_DS_. Then, conductance variation (difference between values before and after the application of *V*_GS_ pulses) was represented as percentage.

Three consecutive measurements were performed, resulting in the sum of 18 applied voltage pulses, and conductance variations were computed and averaged for each device. At least *N* = 3 devices were measured with this procedure to obtain the results shown in the manuscript.

### H_2_O_2_ static measurements and data analysis

H_2_O_2_ static measurements were carried out as described in Methods (DA static measurements and data analysis). The only difference in the procedure is that one measurement with DA (6 voltage pulses) was performed before the application of H_2_O_2_.

### Data analysis

Data analysis was carried out through custom made MATLAB scripts.

### Numerical simulations

Numerical simulations were carried out through COMSOL Multiphysics 6.0, by coupling “laminar flow” and “transport of diluted species” built in modules.

### Robotic hand printing and electrical actuation

The hand was 3D printed using an open-source cable-driven design (InMoov, https://inmoov.fr/). Hand closure was achieved by driving servomotors (one per fingers). Cables were mounted on the motors so that the 0-degree position corresponds to completely open hand, while 90-degree position corresponds to a fully closed hand. Commands were sent to the servomotors through a commercial microcontroller board (Arduino Uno board). A custom-made Arduino script was used to read data through serial interface from the electrical measurement setup and drive the servomotors. When performing reinforcement learning, a feedback signal was sent by serial interface to punish or reward ENODe (Fig. S4 and S5, ESI†).

## Conclusions

Neuromorphic systems featuring innovative materials are emerging in the never-ending quest for the next generation of hardware. In this sense, closed-loop processing is essential, as it represent the foundation of countless real-time and real-world tasks. In light of this, closed-loop neuromorphic system are advisable, as they could potentially lead to fully autonomous and adaptive systems. Here, we presented a simple and direct approach to endow long-standing closed-loop architectures with neuromorphic capabilities. Organic synaptic devices emerged as unique and singular option in such approach, as they can communicate with silicon-based technologies, while providing adaptive and smart features, ultimately leading to the complete delocalization of the intelligence of the system. As a result, while a microcontroller dealt with simple read/write operation through serial interfaces, the organic chips adapted to complete the required task. In perspective, by reducing the size of the organic neuromorphic devices and optimizing the geometrical ratio (W/L),^6,40^ the switching speed of the devices could be reduced, allowing to match biologically plausible operating speed. In addition, as the adaptation closely mirrors biological potentiation/depression paradigms, and it is based on biologically relevant signals, such approach may represent the first step towards smart prosthetics devices and adaptive clinical approaches.

## Author contributions

U. B and D. R. contributed to the experimental design, data collection, analysis, and manuscript writing. C. A, A. M., S. M., O. B., C. L. supported experimental design, F. S. contributed to project and experimental design, data discussion, manuscript revision and funding acquire.

## Conflicts of interest

There are no conflicts to declare.

## Supplementary Material

MH-011-D3MH02202A-s001

MH-011-D3MH02202A-s002

MH-011-D3MH02202A-s003
