# Supplementary material for: An organic brain-inspired platform with neurotransmitter closed-loop control, actuation and reinforcement learning
Source: Mater Horiz. 2024 Apr 30;11(12):2865–74. doi: 10.1039/d3mh02202a (PMC11182378; doi:10.1039/d3mh02202a)
Supplement: MH-011-D3MH02202A-s001 [file MH-011-D3MH02202A-s001.pdf]

Electronic Supplementary Material (ESI) for Materials Horizons.
This journal is © The Royal Society of Chemistry 2024

Supplementary Information

Supplementary Discussion S1: Chemical reactions of dopamine and H_2O_2 with PEDOT:PSS and synaptic plasticity of ENODes.

In biological synapses, an action potential (AP) is generally transmitted across a synapse eliciting the release of neurotransmitter in the synaptic cleft, that evokes a post synaptic potential (PSP) in the post-synaptic neuron[1]. In addition, synapses are not a static structure as they can exhibit synaptic plasticity, *i.e.*, the communication between two neurons can be strengthen/weakened in response to the increase/decrease in the synaptic activity[1], resulting in synaptic potentiation/depression.

Such synaptic events were recapitulated in an ENODe featuring both gate and channel made of PEDOT:PSS, by applying square voltage pulses at the gate terminal (V_{GS}), mirroring the biological presynaptic action potentials.

Such voltage bias caused ions to migrate from the electrolyte to the polymeric channel, mimicking the electrochemical signal transduction of biological synapses, in which APs caused the release of the neurotransmitter in the synaptic cleft. Consequently, the transistor channel was de-doped, changing its conductance level, and resulting in a modulation of the channel current (I_{DS}), that represented a PSP.

Such artificial synaptic communication was volatile, as the removal of the voltage bias at the gate caused ions to migrate back to the electrolyte, restoring the initial charge equilibrium.

Non-volatile potentiation and depression (*i.e.*, synaptic plasticity) of the ENODe was demonstrated by exploiting DA and H_2O_2, respectively.

Here, the application of the characteristic NTs oxidation potential at the ENODe gate terminal induced the release of reducing agents on the PEDOT: PSS layer[2]. The dopamine (DA) was oxidized to dopamine o-quinone (DQ) with the application of pulse trains of 0.3V amplitude:

$DA \rightarrow DQ + 2e^{-} + 2H^{+}$

The cationic species from the electrolyte and the protons produced from the oxidation compensated the anions of the PSS^{-} group, while the electrons eliminated the holes in the $PEDOT^{+}$. This reaction resulted in the depletion of the organic layer and the consequent de-doping:

$PEDOT^{+}: PSS- + e^{-} + Cat^{+} \rightarrow PEDOT^{0} + PSS^{-}: Cat^{+}$

where $PEDOT^{+}$ indicated the pristine state of the polymer and $PEDOT^{0}$ stays for the de-doped state of the polymer.

In order to oxidize (the reduced) PEDOT:PSS, oxygen was shown to be effective[3] H_2O_2 was used as oxidant agent and different concentrations were investigated to obtain a partial recovery of the doping level of the PEDOT:PSS. According to theoretical simulations, a shuttle reaction in the electrolyte between two layers of PEDOT:PSS happens, where a local dissociation of hydrogen peroxide is converted in water and oxygen with the consequent electronic charge transfer in the polymeric channel[4]. We propose that a surface oxidation of the complex PEDOT:PSS-DA occurred in presence of H_2O_2. This would suggest that only a partial recovery happened, not allowing for a complete reversibility of the doping process at the bulk of the organic layer.

Supplementary Figure S1: Sensitivity range of the ENODe upon repeated oxidation of dopamine.

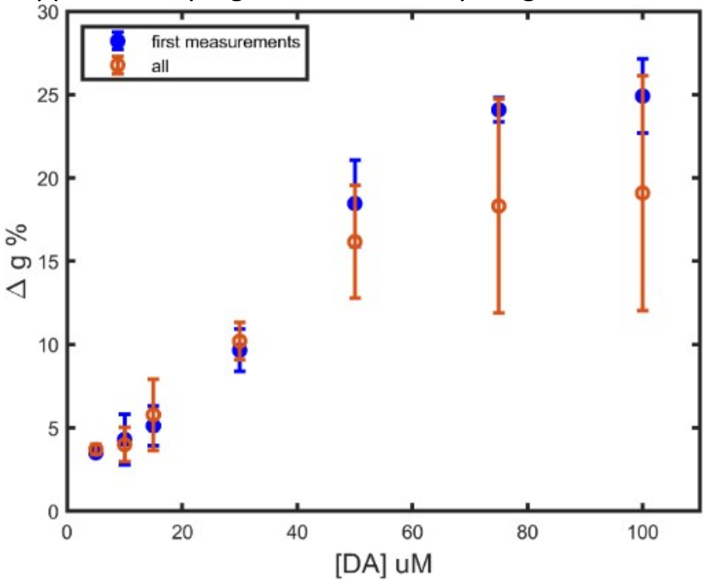

Fig. S1, For the open circuit characterization, different concentrations of dopamine were tested and three measurements for each concentration were repeated for three different devices (N=3, 9 measurements in total). The statistics of the channel conductance variations resulting from the first dopamine measurements for each device and the statistics resulting from all performed measurements were reported. For low concentrations of dopamine solutions ([5,10,15,30] µM), the effect due to the first measurement was comparable with the following measurements performed with the same device in terms of values and error bars (differences < 2%). For high dopamine concentrations ([50,75,100] µM), the PEDOT:PSS channel was saturated, as the mean value of conductance variation decreased after the initial oxidation of neurotransmitter (differences > 2%).

Supplementary Figure S2: Recovery of the ENODe with H_2O_2 after different concentrations of dopamine.

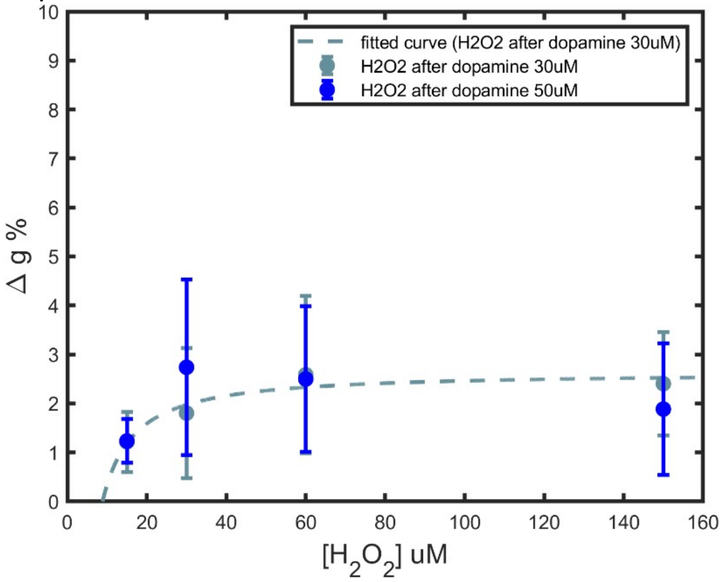

Fig. S2, Channel conductance recovery due to different H_2O_2 concentration after doping PEDOT:PSS with dopamine solutions (30 mM and 50 mM). In both cases, the percentage variation of conductance did not increase with H_2O_2 concentrations higher than 60 µM, showing a saturation of the recovery effect . The figure also reports the measured conductance variations which were independent of the neurotransmitter concentration and de-doping level of the PEDOT: PSS prior to measurements, suggesting that the recovery mechanisms might be related to reactions occurring at the surface of the de-doped PEDOT: PSS only (Supplementary Information S1). This would also support the saturation effect of the oxidation of the organic polymer, regardless of its level of de-doping.

Supplementary Figure S3: Control law in a PID compensator.

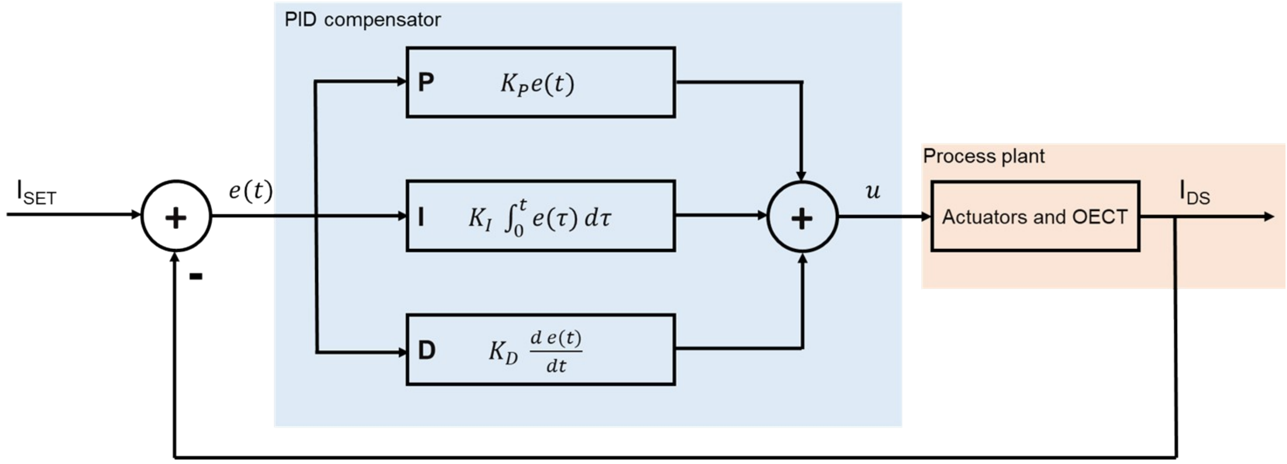

Fig. S3, In a conventional feedback control system, the process is the object to be controlled and the compensator makes the process variable (I_{DS}) follow the setpoint value (I_{SET}). The compensator drives change a variable (u). The error (e(t)) is the input variable of the compensator C(t) that is the computational rule used to manipulate u. A PID compensator is characterized by the proportional, integrative, and derivative parameters that describe three different contributions to reduce the error.

The manipulated variable u can be written in function of three constant (K_P, K_I , K_D) to weight the corresponding contributes of the control system described in Fig. S5:

$$u(t) = K_P e(t) + K_I \int_0^t e(\tau) d\tau + K_D \frac{de(t)}{dt}$$

In this work the parameters used were: $K_P = 0.005; K_I = 0.05; K_D = 0.01.$ These values were adjusted by considering the response of the system to the different variations.

Supplementary Figure S4: Connections between the robotic hand, Arduino board and measurement set-up.

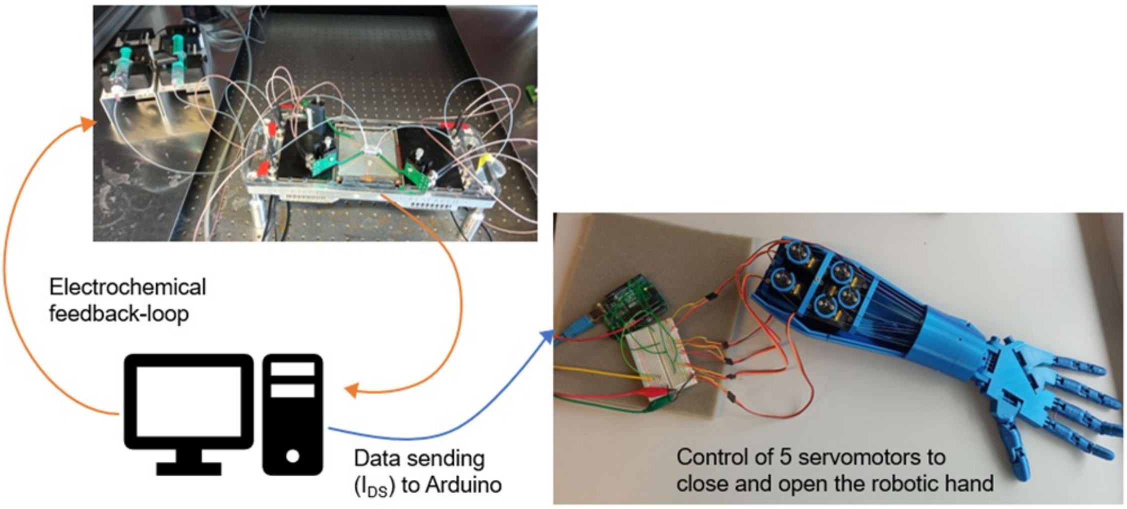

Fig. S4, Schematics of connections of the electrochemical feedback-loop and of the robotic hand. The channel current value of the ENODe was recorded and a response signal was sent to the microfluidic system (left), controlled by the PID control software. After the application of each voltage pulse, the channel current value was sent to Arduino, which controlled five servomotors, assembled in the robotic hand (right). To this end, Arduino was connected to customized PID software (Arkeo Cicci Research srl) and the different motors were connected to the five analogic outputs of Arduino through a board. Each motor was connected through nylon thread to a single finger. When an electrical signal was applied, the angle of the motors varied, resulting in the wire pulling of the fingers.
The servomotors were powered by a 7V supplier.

Supplementary Discussion S2: Operation of the feedback-loop control system.

The variable controlled by the feedback-loop control system was the channel current amplitude of the ENODe after the application of a square voltage pulse at the gate terminal.
The workflow of the customized software based on lab view is the following:

1) Prior to the initiation of the experiment, these parameters were set as follows:
   - the value of the drain constant voltage V_{DS} was - 0.2V;
   - the shape of the pulse to be applied as voltage gate was defined with 2s of ON time duration, 6s of OFF time and 0.3V amplitude;
   - the microfluidic pump that should work depending on the status of the controller was chosen by selecting the sign of the error. For positive errors, the pump with the dopamine solution was activated whereas for negative errors the other pump would be activated.
   - the maximum flow rate that the pumps can reach was set to 1 mL/min;
   - the first desired value for channel current I_{SET} was chosen in the tolerance regime of the device (known from the electrical characterization of the ENODe) and the range of error was set in the order of µA.
2) As the experiment started the microfluidic channel of the ENODe contained PBS solution only. The first pulse was applied to the gate and the channel current I_{DS} was measured after the removal of the pulse.
3) The error was calculated as: $e(t) = I_{SET} - I_{DS}$, where both I_{DS} and I_{SET} had negative values.
   - if $I_{SET} > I_{DS}$, a positive error resulted and the pump for the dopamine solution was initiated with a flow rate that depended on the amplitude of the error and the set values for the proportional, integrative and derivative constants of the PID (as described for the control law in Supplementary Information S5);
   - if $I_{SET} < I_{DS}$, a negative error was measured and the pump for the H_2O_2 solution was activated with a flow rate depending on the error value and the set values for the proportional, integrative and derivative constants of the PID (as described for the control law in Supplementary Information S5).
4) Depending on the OFF time selected of the gate voltage, a new pulse was applied to the ENODe, inducing a variation of I_{DS} associated with the oxidation of dopamine or the action of H_2O_2. The workflow was then repeated starting from point (2), considering that I_{SET} could be modified any time during the experiment.

Supplementary Figure S5: Experiment of reinforcement learning and electrical-closed loop on the robotic hand.

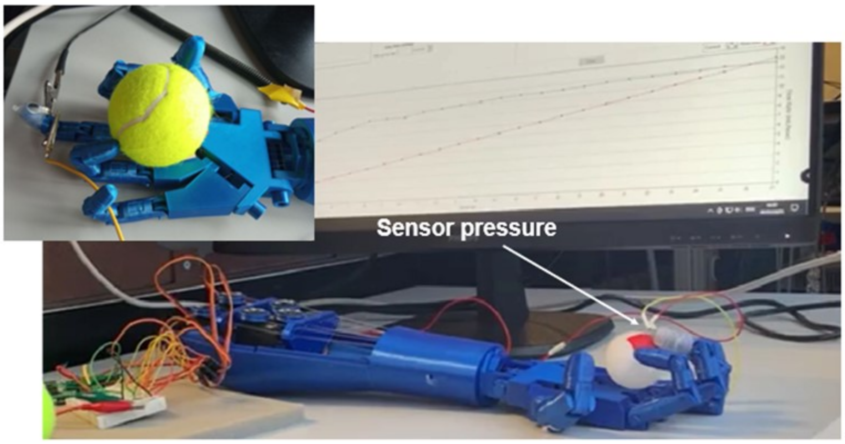

Fig. S5, Picture of the robotic hand and the sensor pressure placed on the external phalanx of the middle finger, to detect the gripping of the ball. In this work, the thin pressure sensor was combined with a 10kΩ resistor, to obtain a variable voltage that could be read by a microcontroller's analog-to-digital converter when a force was applied.

To evaluate the reinforcement learning capability of the ENODe coupled to the robotic hand, the customized software based on LabVIEW followed this workflow:

0) Prior to the initiation of the experiment, these parameters were set as follows
   - the value for the drain constant voltage V_{DS} was - 0.2V;
   - the shape of the pulse to be applied as voltage gate was defined with 2s of ON time duration, 6s of OFF time and 0.3V of amplitude;
   - the dopamine solution pump was activated.
   - the maximum flow rate that the pumps could reach was set to 0.2 mL/min, lower than in the experiments of characterization for the control to avoid unnecessary dopamine solution usage during long measurements (~ 6-8 minutes);
   - in this experiment the PID controller allowed a continuous flow of dopamine until the electrical control from the pressure sensor was reached or the PEDOT:PSS channel of the ENODe is not completely de-doped, then the desired value for channel current I_{SET} is –10 µA (~0 µA) and the range of error is set in the order of µA.
   - a delay time of 10s was set to allow Arduino to process the measured data without interferences with the measurement system.
   - a threshold th_start = 10 µA was set in the Arduino script to indicate for which modulation of the channel current I_{DS} the robotic hand should start the closure movement.
   - a threshold th_end = 40 µA was defined in the Arduino script to indicate for which values of current modulation the robotic hand should be completely closed. In this way, the different motor angles are mapped on a range of channel currents of the ENODe.
1) As the experiment started, the microfluidic channel of the ENODe contains PBS solution only. After the delay time, the first pulse was applied to gate and the channel current I_{DS} after the removal of the pulse was measured.
2) The error was calculated as: $e(t) = I_{SET} - I_{DS}$, where both I_{DS} and I_{SET} reached negative values.
   - In this experiment $I_{SET} > I_{DS}$ and a positive error was obtained: the pump for the dopamine solution was activated with a flow rate that depended on the amplitude of the error and the set values for the proportional, integrative and derivative constants of the PID (as described for the control law in Supplementary Information S5).
3) When the value of I_{DS} reached the th_start, the motors of robotic hand rotate.

The sensor pressure measurement was then monitored:
- if its value was 0, the cycle was repeated and the hand opened again (learning process);
- If its value was higher than 0 (some force was applied), the motors would stop in the position they were until the force was removed and the learning process (for the position) was finished.

Supplementary Figure S6: Electrical characterization of the steady-state behaviour of ENODes

The steady state behaviour of ENODEs was characterized to determine the range of operation and transconductances achieved from the devices used for the neuromorphic platform. In **Fig.S6** the output curves of the device were reported. The range of operation is in the order of 10^{-5} 10^{-4} A and for this reason the desired values of current for the closed-loop were in this range of operation. Also the thresholds values for the closure and opening of the robotic hand was chosen according to this results.

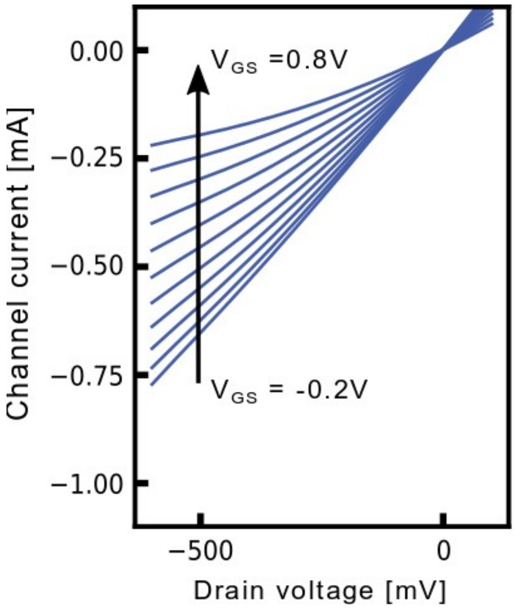

Fig. S6, Channel current of ENODes when different values of V_{DS} [-0.6 V ,0.1 V] were applied, for different values of V_{GS} [-0.2 V, 0.8 V], showing a switching off the device for highest values of gate voltage.

References

1. Kandel, E. R., Schwartz, J. H., Jessell, T. M., Siegelbaum, S. A. & Hudspeth, A. J. *Principles of Neural Science*. vol. 4 (McGraw-hill New York, 2000).
2. Keene, S. T. *et al.* A biohybrid synapse with neurotransmitter-mediated plasticity. *Nat. Mater.* **19**, 969–973 (2020).
3. Singh, G., Kushwaha, A. & Sharma, M. Highly sensitive and selective detection of serotonin and dopamine with stable oxidation potentials using novel Dy2MoO6 nanosheets. *Materials Chemistry and Physics* **279**, 125782 (2022).
4. Felder, D. *et al.* Coupled Ionic–Electronic Charge Transport in Organic Neuromorphic Devices. *Advcd Theory and Sims* **5**, 2100492 (2022).
